# The Willingness to Intervene in Cases of Intimate Partner Violence Against Women (WI-IPVAW) Scale: Development and Validation of the Long and Short Versions

**DOI:** 10.3389/fpsyg.2018.01146

**Published:** 2018-07-17

**Authors:** Enrique Gracia, Manuel Martín-Fernández, Miriam Marco, Faraj A. Santirso, Viviana Vargas, Marisol Lila

**Affiliations:** Department of Social Psychology, University of Valencia, Valencia, Spain

**Keywords:** intimate partner violence, violence against women, willingness to intervene, public attitudes, intervention preferences, help-giving, bystander intervention, measurement

## Abstract

Willingness to intervene when one becomes aware of a case of intimate partner violence against women (IPVAW) reflects the level of tolerance and acceptance of this type of violence in society. Increasing the likelihood of intervention to help victims of IPVAW is also a target for prevention strategies aiming to increase informal social control of IPVAW. In this study, we present the development and validation of the Willingness to Intervene in Cases of Intimate Partner Violence (WI-IPVAW) scale. We report data for both the long and short versions of the scale. We analyzed the latent structure, the reliability and validity of the WI-IPVAW across four samples (*N* = 1648). Factor analyses supported a bifactor model with a general non-specific factor expressing willingness to intervene in cases of IPVAW, and three specific factors reflecting different intervention preferences: a preference for setting the law enforcement process in motion (“calling the cops” factor), a preference for personal intervention (“personal involvement” factor), and a preference for non-intervention (“not my business” factor). Configural, metric, and partial scalar invariance across genders were supported. Two short versions of the scale, with nine and six items, respectively, were constructed on the base of quantitative and qualitative criteria. The long and short versions of the WI-IPVAW demonstrated both high reliability and construct validity, as they were strongly related to the acceptability of IPVAW, victim-blaming attitudes, perceived severity of IPVAW, and hostile sexism. These results confirm that both the long and short versions of the WI-IPVAW scale are psychometrically sound instruments to analyze willingness to intervene in cases of IPVAW in different settings and with different research needs (e.g., long versions for clinical and research settings, and short versions for large population surveys). The WI-IPVAW is also useful for assessing prevention policies and public education campaigns design to promote a more responsive social environment in cases of IPVAW, thus contributing to deter and reduce this major social and public health problem.

## Introduction

The World Health Organization defines intimate partner violence against women (IPVAW) as a “global public health problem of epidemic proportions” (World Health Organization [WHO], 2013, p. 7). IPVAW has profound consequences not only for the physical and psychological health of victims, but also for the well-being of their children, and for society in general (e.g., Campbell, 2002; Ellsberg et al., 2008; Devries et al., 2011; World Health Organization [WHO], 2013; Guedes et al., 2016). IPVAW is considered the most common form of violence suffered by women (Garcia-Moreno et al., 2006; Devries et al., 2013; Stöckl et al., 2013). In high-income countries, the estimated prevalence of IPVAW is 23.2%, and the percentage of IPVAW homicides, 41.2% (World Health Organization [WHO], 2013). In Europe, a survey among the 28 European Union (EU) Member States estimated that an average of 22% of European women had been victims of physical and/or sexual violence by their partners since the age of 15, with a lifetime prevalence across countries ranging from 13 to 32% (European Union Agency for Fundamental Rights, 2014). In Spain, where this study was conducted, various sources estimate IPVAW lifetime prevalence at around 13%, among the lowest in the EU (Vives-Cases et al., 2011; European Union Agency for Fundamental Rights, 2014; Ministerio de Sanidad, Servicios Sociales e Igualdad, 2015; Gracia and Merlo, 2016).

An ecological model recognizes that beyond individual and relational explanatory levels, larger contextual and societal factors are central to understand IPVAW (Heise, 1998, 2011; World Health Organization [WHO], 2002; Gracia et al., 2015a). As Gracia and Lila (2015, p. 16) pointed out, ‘violence against women is a complex phenomenon that needs to be understood within the wider social context and within the social and cultural norms that permeate it.’ Public attitudes toward IPVAW shape the social context in which IPVAW takes place and play an important role in perpetuating the levels of this type of violence in our societies (Carlson and Worden, 2005; Flood and Pease, 2009; Waltermaurer, 2012; Gracia and Lila, 2015; Copp et al., 2016; Powell and Webster, 2018). Public willingness to intervene when one becomes aware of a case of IPVAW reflects the level of tolerance and acceptance of this type of violence and can contribute either to deter or facilitate it (Browning, 2002; Gracia and Herrero, 2006; Emery et al., 2011; Wright and Benson, 2011; World Health Organization [WHO], 2013; Jewkes et al., 2015). In the current study, we set out to develop a scale measuring public willingness to intervene in cases of IPVAW.

One reason for studying willingness to act in cases of IPVAW is that, despite still being a largely unreported offense, at the same time IPVAW is widely known in the victims’ social environment (Gracia, 2004; Taylor and Sorenson, 2005; Taylor et al., 2016). For example, in a survey across the 28 European Union member states, nearly 23% of respondents reported knowing a woman among their family members or friends who had been victim of IPVAW, 17% reported knowing women in their immediate neighborhood, and 9% knew a woman where they worked or studied (European Commission, 2016). Those who are aware of IPVAW incidents are in a position to do something to help the victims and stop the violence (e.g., offering help, taking personal action, or setting the law in motion), but they can also choose not to get involved, to ignore the situation, and do nothing (Banyard and Moynihan, 2011; Taylor et al., 2016). Therefore, whether or not those who are aware of this violence are willing to intervene is a not a trivial matter.

Attitudes of non-intervention in the victim’s social circle may facilitate or reinforce the perpetrator’s behavior, but may also inhibit victims’ disclosure, making it more difficult for them to seek help and escape the violence. On the other hand, pro-intervention attitudes (e.g., reporting to the authorities or direct intervention) among those aware of this violence can have a protective effect for victims, and may inhibit or deter IPVAW by increasing the social and legal costs for perpetrators (Koepsell et al., 2006; McDonnell et al., 2011; Gracia, 2014; Voith, 2017). Willingness to intervene among those who are aware of IPVAW incidents is also relevant because victims tend to seek help among informal sources of help (friends, family, neighbors, coworkers, etc.) rather than formal sources such as the police (Liang et al., 2005; Ansara and Hindin, 2010; McCart et al., 2010; McDonnell et al., 2011; Wee et al., 2016). Moreover, pro-intervention attitudes among these potential informal sources of help, when shared collectively, can contribute to shape local social norms that help deter this type of violence (Wee et al., 2016; Voith, 2017; Powell and Webster, 2018). As Voith (2017, p. 4) noted in her review, “the protective effects of pro-IPV-intervention norms in a community are twofold, in that community members will directly intervene if they witness IPV and perpetrators are less likely to continue the use of violence against their partners as a result of social pressure”.

Another reason to study and accurately measure public willingness to act in cases of IPVAW is that evidence suggests non-intervention attitudes are still quite prevalent, as shown in one report on attitudes toward violence against women in the EU (Gracia and Lila, 2015). For example, data from surveys carried out in different countries indicate that a sizable number of respondents preferred not to get involved even if they were aware of a case of violence against women (“not my business,” or “is a private matter” were among the reasons given for not intervening). In addition, across the EU (European Commission, 2016), the most common reason given by those who knew victims of domestic violence but did not speak about it to anyone was that it was “none of their business” (26%). “Lack of proof” (18%), “not wanting to create trouble” (16%), “concerned about negative consequences or retaliation” (11%), “did not know who to speak to” (8%), and “it was not serious enough” (6%), were some other reasons. In Spain, where the present study was conducted, most of the officially reported cases of IPVAW are made by the victims themselves, and only around 4% of such reports come from family members or other third parties (Consejo General del Poder Judicial, 2016). Increasing the likelihood that people will intervene to help victims of IPVAW is therefore a target for prevention strategies aiming to translate public awareness of this social problem into a greater sense of personal responsibility and involvement, thus contributing to the informal social control of IPVAW (Gracia et al., 2009).

### Present Study

Drawing from the above, there is an evident need to advance our knowledge about public willingness to intervene in cases of IPVAW and related key issues such as the prevalence of pro- or non-intervention attitudes, intervention preferences, its correlates or determinants, or assessing the effectiveness of interventions targeting these attitudes. The availability of reliable and valid instruments measuring public willingness to intervene in cases of IPVAW is central to this type of research. Although some measurement instruments have been developed to examine willingness to help in cases of violence, most of this research has been conducted in the context of bystander intervention behavior in cases of dating violence, and sexual harassment or rape situations (Stein, 2007; Banyard, 2008; Banyard and Moynihan, 2011; Branch et al., 2013; Banyard et al., 2014; McMahon et al., 2014). Other studies assessing willingness to intervene have limited generalizability as they use small non-community samples (e.g., college students), and others instruments report low reliabilities (Baldry and Pagliaro, 2014; Baldry et al., 2015; Cinquegrana et al., 2018). In addition, data from large population surveys on public attitudes toward intervention in cases of IPVAW are not usually based on measurement instruments with adequate reliability and validity, or rely on single items (Gracia and Lila, 2015). Clearly, there is still a need for psychometrically sound instruments measuring willingness to intervene in cases of IPVAW, appropriate for use with community samples, and suitable for large-scale surveys.

In this study, we present the development and validation of the Willingness to Intervene in Cases of Intimate Partner Violence (WI-IPVAW) scale. We aim also to develop reduced versions of the full WI-IPVAW scale, as large population surveys or studies with limited space or time require the use of short forms while retaining adequate psychometric properties (Smith et al., 2000; Goetz et al., 2013). By reporting data for both the long and short versions of the scale, we aim to provide tools to analyze willingness to intervene in cases of IPVAW in different settings and with different research needs (e.g., long versions for clinical and research settings, and short versions for large population surveys). By using advanced statistical analyses, we will address important issues such as social desirability and measurement invariance and ensure that the shortened versions of the WI-IPVAW scale retain high quality psychometric properties.

For validity purposes, we will explore the relationship between the long and short versions of the WI-IPVAW scale and other relevant constructs regarding attitudes toward IPVAW such as IPVAW acceptability, victim-blaming attitudes, perceived severity of IPVAW, and hostile sexism (Taylor and Sorenson, 2005; Gracia and Herrero, 2006; Flood and Pease, 2009; Lila et al., 2013; Gracia, 2014; Herrero et al., 2017; Martín-Fernández et al., 2018b). Gender, age and education differences in willingness to intervene in cases of IPVAW will be also explored (Carlson and Worden, 2005; Fincham et al., 2008; Flood and Pease, 2009; Gracia et al., 2009; Gracia et al., 2015b). Attitudes of acceptability of IPVAW have been considered a key issue to understand IPVAW prevalence in society (Flood and Pease, 2009; Gracia et al., 2015b; Copp et al., 2016; Martín-Fernández et al., 2018b). These attitudes have been linked to public, professionals, and victims’ perceptions and responses to IPVAW (Taylor and Sorenson, 2005; Gracia and Herrero, 2006; Rizo and Macy, 2011; Gracia et al., 2014). We hypothesize that the lower the IPVAW acceptability, the greater the willingness to intervene in cases of IPVAW. Victim-blaming attitudes are also among those factors often used to explain and justify IPVAW. These attitudes can influence public responses toward known cases of IPVAW (Liang et al., 2005; Ansara and Hindin, 2010; Gracia, 2014; Gracia and Tomás, 2014). We expect that lower scores of victim-blaming attitudes will be associated with greater willingness to intervene in cases of IPVAW. The perceived severity of IPVAW incidents may also influence responses to IPVAW (Gracia et al., 2009, 2014). According to Latané and Darley’s (1970) model of bystander intervention, perceived severity is a precondition to the decision to intervene. According to this model, if some incidents of IPVAW are perceived as not serious enough, bystanders will be less willing to intervene (Gracia et al., 2009). We anticipate that the greater the perceived severity of IPVAW, the greater the willingness to intervene in cases of IPVAW. Hostile sexism is a gender prejudice manifestation that conveys negative images and beliefs about women (Glick and Fiske, 1996), and has been related to attitudes toward intervention in cases of IPVAW (Lila et al., 2013; Herrero et al., 2017). We hypothesize that the lower the hostile sexism, the greater the willingness to intervene in cases of IPVAW. Finally, gender, age and education differences in willingness to intervene in cases of IPVAW will be also explored (Carlson and Worden, 2005; Fincham et al., 2008; Flood and Pease, 2009; Gracia et al., 2009, 2015b).

## Materials and Methods

### Participants

Four samples were recruited for the current study. The first one was an incidental sample used to conduct a pilot study, composed of 148 Valencia University undergraduates who participated for course credits (31 males and 117 females), aged 19–32 years old (*M* = 21.29; *SD* = 2.60). The second, third, and fourth samples were recruited through online sampling. Online sampling is an effective and cost-efficient sampling method (Thornton et al., 2016; Topolovec-Vranic and Natarajan, 2016). A total pool of 2,698 responses was collected. We equilibrated these samples by gender and removed those participants who were younger than 18 years old, omitted socio-demographic information, or were duplicated responses. Participants from samples 2, 3, and 4 were randomly drawn from the remaining pool of responses. The socio-demographic characteristics of the samples are shown in **Table 1**.

**Table 1:** Socio-demographics.

	Sample 1	Sample 2	Sample 3	Sample 4
**Gender**				
Male	117 (79.1%)	231 (46.2%)	510 (51.0%)	94 (47.0%)
Female	31 (20.9%)	269 (53.8%)	490 (49.0%)	106 (53.0%)
**Age**				
18–24	131 (88.5%)	214 (42.8%)	243 (24.3%)	108 (54.0%)
25–34	14 (9.5%)	83 (16.6%)	311 (31.1%)	30 (15.0%)
35–54	2 (1.3%)	141 (28.2%)	347 (34.7%)	53 (26.5%)
55+	1 (0.7%)	62 (12.4%)	99 (9.9%)	9 (4.5%)
**Nationality**				
Spanish	128 (86.5%)	429 (85.8%)	869 (86.9%)	186 (93.0%)
Inmigrant	20 (13.5%)	61 (14.2%)	131 (13.1%)	14 (7.0%)
**Education**				
Compulsory	0	65 (13.0%)	143 (14.3%)	25 (12.5%)
Upper secondary	0	88 (17.6%)	191 (19.1%)	38 (19.0%)
Undergraduate	135 (91.2%)	190 (38.0%)	321 (32.1%)	89 (44.5%)
Postgraduate	13 (8.8%)	157 (31.4%)	345 (34.5%)	48 (24.0%)


The second sample consisted of 500 participants (231 males and 269 females), aged 18–80 (*M* = 33.83; *SD* = 14.77), and was used to study the psychometric properties of the scale. The third sample consisted of 1000 participants (490 males and 510 females), aged 18–82 (*M* = 35.40; *SD* = 13.46). This sample was used to test different levels of measurement invariance and to conduct the criterion-related validity analyses. The fourth sample consisted of 200 participants (94 males and 106 females), aged 18–71 (*M* = 29.39; *SD* = 11.82), and was used to assemble two short versions of the scale.

### Measures

#### Willingness to Intervene in Cases of IPVAW (WI-IPVAW)

The development of the WI-IPVAW was based on an initial pool of 96 items. These items were developed from a review of European surveys addressing attitudes toward intervention in cases of violence against women (Gracia and Lila, 2015), and other previous research addressing public attitudes and response preferences in cases of IPVAW (Gracia and Herrero, 2006; Gracia et al., 2009). The item development and selection process was also informed by literature identifying scenarios where IPVAW also takes place, other than behind closed doors at home, and is witnessed by third parties (Banyard and Moynihan, 2011; Hamby et al., 2015; Taylor et al., 2016). This initial pool of items presented hypothetical scenarios describing IPVAW situations, occurring in different places, and that could be witnessed by the respondent, or disclosed to him/her by the victim (e.g., next door apartment, staircase or communal areas in buildings, street, shops, bars, etc.). These scenarios included various expressions of IPVAW behaviors (e.g., physical aggression, insults, threats, violent arguments, fights, etc.), and different types of potential responses or involvement (i.e., calling the police, scolding or reprehending the aggressor, protecting the woman victim, ignoring the situation, doing nothing, etc.). The initial pool of items was then reviewed by a panel of six experts on IPVAW to establish construct representativeness and clarity (Beck and Gable, 2001; Delgado-Rico et al., 2012). The experts were asked to rate the representativeness (i.e., whether the item is suitable to measure willingness to intervene in cases of IPVAW), and the clarity (i.e., how concise the item is) of the items on a 7-point Likert-type scale (1 = “Very unrepresentative/unclear”; 7 = “Very representative/clear”). An item was considered representative and/or clear if the average score in the expert ratings was above 5 on the 7-point scale (i.e., the “somewhat representative/clear” category). After this review, 31 items were selected. Respondents were asked to rate their perceived likelihood of intervening in the hypothetical scenario described in each item on a 6-point Likert-type scale (1 = “Not at all likely,” 6 = “Extremely likely”). The final version of the WI-IPVAW scale is shown in Appendix 1 (see Supplementary Material).

#### Acceptability of IPVAW (A-IPVAW; Martín-Fernández et al., 2018b)

The short form of the A-IPVAW scale was used in this study. This instrument is composed of eight items tapping attitudes of acceptability of IPVAW (e.g., It is acceptable for a man “to shout his partner if she is continuously arguing and nagging him”). Respondents rated the acceptability of a range of men’s behaviors against their female partners on a 3-point Likert-type scale (0 = “Not acceptable,” 1 = “Somewhat acceptable,” 2 = “Acceptable”). The A-IPVAW scale was cross-validated in the general Spanish population, and also with IPVAW male offenders. This scale has showed adequate internal and external validity, as it has been related to perceived severity of IPVAW and ambivalent sexism (Martín-Fernández et al., 2018b). Our results showed reasonable internal consistency across Samples 2, 3, and 4 (Cronbach’s α = 0.75, 0.72, 0.68, respectively).

#### Victim-Blaming Attitudes Toward IPVAW (VB-IPVAW; Martín-Fernández et al., 2018a)

This instrument is composed of five items assessing the tendency to blame victims of IPVAW (e.g., “A man will change his behavior toward his partner if she becomes more obedient”). Respondents rated their level of agreement with each statement on a 4-point Likert-type scale (1 = “Strongly disagree,” 4 = “Strongly agree”). Evidence of the instrument’s validity has been demonstrated based on its relationships with other variables such as the acceptability and perceived severity of IPVAW, and ambivalent sexism (Martín-Fernández et al., 2018b). It also presented high internal consistency in Samples 2, 3, and 4 (Cronbach’s α = 0.81, 0.84, 0.83, respectively).

#### Perceived Severity of IPVAW (PS-IPVAW; Gracia et al., 2009, 2011)

This scale presents eight IPVAW scenarios (e.g., “During an argument, a man hits his partner and then asks her to forgive him”), the severity of which respondents assessed on a 10-point Likert-type scale (ranging from 1, “Not severe at all,” to 10, “Extremely severe”). The PS-IPVAW scale has previously been validated in the general Spanish population, and also with police officers and male IPVAW offenders, presenting adequate psychometric properties. It has also been related to sexism, empathy, personal responsibility, and IPVAW victim-blaming attitudes (Gracia et al., 2009; Lila et al., 2013; Gracia and Tomás, 2014; Vargas et al., 2015). The scale showed good internal consistency in Samples 2, 3, and 4 (Cronbach’s α = 0.83, 0.85, 0.87, respectively).

#### Ambivalent Sexism Inventory Short Version (ASI; Glick and Fiske, 1996; Rollero et al., 2014)

The reduced hostile sexism subscale was used for the current study, composed of six items assessing attitudes of prejudice and discrimination against women based on the assumption of women’s inferiority and their differences from men (e.g., “Women seek to gain power by getting control over men”). The Spanish version of the items was used (Expósito et al., 1998). The complete ambivalent sexism inventory has been validated in more than twenty countries (Glick et al., 2000, 2002), and the hostile sexism subscale has demonstrated strong relationships with attitudes toward intervention in IPVAW cases among police officers, IPVAW responsibility attribution, and acceptability of IPVAW (Lila et al., 2013, 2014; Martín-Fernández et al., 2018b). It presented good internal consistency in Samples 2, 3, and 4 (Cronbach’s α = 0.89, 0.88, 0.87, respectively).

#### Balanced Inventory of Desirable Responding Short Form (BIDR-16; Hart et al., 2015)

The Impression Management subscale was used for the pilot study. This subscale is composed of eight items evaluating the tendency of participants to provide overestimated self-descriptions to create a socially desirable image (e.g., “I never cover up mistakes”), and presented moderate reliability in the first sample (Cronbach’s α = 0.68).

### Procedure

Two online forms were designed to collect the data. The first form included the WI-IPVAW, the BIDR items of the Impression Management subscale, and a set of socio-demographical questions (i.e., gender, age, nationality, and education level). This form was used only for Sample 1. Participants were informed about the objectives of the study and gave their informed consent, agreeing to participate in the study if they press the “continue” button. The second form included the WI-IPVAW, the PS-IPVAW, the short forms of the A-IPVAW, VB-IPVAW, Hostile Sexism, and the same socio-demographical questions. After the participants had given their informed consent and agree to participate in the study, they completed the online form. Participants received no payment. The data were collected from October 2017 to December 2017.

### Data Analysis

A pilot study was conducted first using the sample of college students (Sample 1) in order to explore the psychometric properties of the WI-IPVAW and the effect of social desirability on the items. One of the major threats to the content validity of any scale assessing personality traits or attitudinal components is the social desirability bias. This bias is a major concern when the assessment involves socially sensitive issues, as IPVAW (Grimm, 2010). Therefore, the aim of this preliminary evaluation was to refine the instrument before administering it to a larger sample. To this end, the descriptive statistics and the item-test corrected correlations were computed, and the internal consistency of the scale was evaluated by means of Cronbach’s α. The latent structure of the scale was also assessed through an exploratory factor analysis (EFA). Before conducting the EFA, the suitability of the data matrix was tested, computing Bartlett’s sphericity test and the Kaiser-Meyer-Olkin (KMO) statistic. To determine the number of factors to extract, a parallel analysis based on minimum rank factor analysis was conducted (Timmerman and Lorenzo-Seva, 2011). An EFA was then performed using the polychoric correlation matrix and the weighted least-squares means and variances adjusted estimation method (WLSMV), as this procedure is especially recommended for categorical data (Muthén and Kaplan, 1985, 1992; Asparouhov and Muthén, 2010). The fit of the model was assessed using the CFI, TLI, SRMR, and RMSEA fit indices. CFI and TLI values ≥ 0.95 are indicative of very good fit, and values between 0.90 and 0.95 indicate minimally acceptable model fit (Bentler, 1995; Hu and Bentler, 1999). RMSEA values ≤ 0.06, and ≤0.08, indicate very good and acceptable fit, respectively, and SRMR values ≤ 0.08 are considered to reflect well-fitting models (MacCallum et al., 1996). Once the latent structure of the scale had been established, the social desirability of each item was evaluated. To do so, a confirmatory factor analysis (CFA) was conducted with the addition of a social desirability factor to the EFA model. All the items of the BIDR and the WI-IPVAW scale were constrained to load onto this social desirability factor, using the BIDR items as social desirability markers (Ferrando, 2005, 2008). To make the model identifiable, the loadings of the BIDR were fixed to the same value. If a WI-IPVAW item loading on the social desirability factor was greater than the BIDR loadings, we considered the item to be biased by social desirability. Those items were removed from the scale.

A larger sample (Sample 2) was used to study further the psychometric properties of the WI-IPVAW scale and to cross-validate the factorial model. The descriptive statistics, the item-test correlations, and Cronbach’s α were again computed. A CFA was carried out using the WLSMV estimation method. Several nested models were compared. Model fit was evaluated using the same combination of fit indices and the same cut-offs.

Measurement invariance across genders was also evaluated in an independent sample (Sample 3). To this end, several levels of group invariance were tested by conducting and comparing a series of multi-group CFAs. Configural, metric, scalar and strict invariance models were estimated using the WLSMV estimation method (Milfont and Fischer, 2010). Configural invariance tests whether men and women conceptualize the construct in the same manner, estimating the same factorial model for each group and allowing the structural parameters (i.e., loadings, thresholds, and item variances) to vary across groups. The metric invariance model constrains the item loadings to have the same value for both groups, testing whether men and women interpret the items in the same way. The scalar invariance model fixes the threshold parameters to the same value across groups, establishing whether the latent construct yields the same score in the items for men and women. The strict invariance model assesses whether the measurement error is equal in each group, constraining the variances of the observed variables (i.e., the items) to have the same values across groups. The models were compared following the guidelines of Cheung and Rensvold (2002), computing the change in CFI (ΔCFI) and RMSEA (ΔRMSEA) to test which of the invariance models is better supported by the data. A change in the CFI (ΔCFI) and in the RMSEA (ΔRMSEA) ≤ 0.010 and ≤0.015, respectively, support the more restrictive model (i.e., the configural model is the most flexible model and the strict invariance the most restrictive). However, these criteria were proposed for models estimated with maximum likelihood estimation for continuous variables and, given that we used weighted least-squares estimation for categorical data, we also ran a corrected chi-square difference test (DIFFTEST; Asparouhov et al., 2006). If the fit indices comparisons and the DIFFTEST yield a similar result, then that invariance level is accepted.

The validity of the scale was assessed by relating it to other relevant IPVAW variables, namely, acceptability of IPVAW, attitudes of victim blaming in cases of IPVAW, perceived severity of IPVAW, and hostile sexism. Socio-demographic comparisons were also made, testing differences across gender, age, and education level groups.

Finally, two short versions of the WI-IPVAW scale of nine and five items were created following Goetz et al. (2013) recommendations. First, the most relevant items were selected attending to the internal consistency, the previous factorial models, and the assessments of the expert panel. The psychometric properties of the shortened scales were then studied and compared with the original WI-IPVAW scale using a different sample (Sample 4).

All analyses were computed using the statistical package R (R Core Team, 2017) and the *psych* library (Revelle, 2016). EFA, CFA, and multi-group CFAs were conducted with the *MPlus 7.1* package (Muthén and Muthén, 2010).

## Results

### Pilot Study: Factor Structure and Social Desirability

The psychometric properties, the latent structure and the effect of social desirability on the WI-IPVAW items were explored in a pilot study with Sample 1. Descriptive statistics revealed that most of the items were slightly displaced to the right, with means around 3–5 (e.g., “somewhat likely,” “quite likely,” “very likely”), and moderate negative skew (around -0.50), indicating that the participants tended to select the upper categories of the scale. The overall internal consistency of the scale was very high (Cronbach’s α = 0.93), showing a strong relation between the score on the scale and the items, with item-test corrected correlations around 0.50. Deleting items did not improve the scale’s internal consistency.

Before conducting an EFA, the suitability of the matrix for factor analysis was tested. Bartlett’s sphericity test was significant (χ^2^ = 2505.8, *df* = 465, *p* < 0.001) and the Kaiser-Meyer-Olkin statistic was good (KMO = 0.88), indicating that the data were adequate for an EFA. The parallel analysis based on minimum rank factor analysis using the polychoric correlation matrix revealed that three factors should be extracted, since adding more factors did not contribute to explain more variance in our data than in a random dataset. A three-factor model was thus estimated using WLSMV with the oblique OBLIMIN rotation. The model converged normally, and showed an acceptable fit (χ^2^ = 2505.8, *df* = 465; CFI = 0.94; TLI = 0.92; RMSEA = 0.068; SRMR = 0.069). Although the CFI and the TLI were below the 0.95 cut-off, they were not below 0.90, and the RMSEA and SRMR suggested that the model was well-fitted. The items were grouped in three factors. The first factor groups all the items related to setting the law in motion by calling to the police or reporting the IPVAW incident (i.e., “calling the cops” factor), the second factor groups all items referring to ignoring the situation or doing nothing (i.e., “not my business factor”), and the third factor groups all items in which the respondents personally intervene to stop the situation (i.e., “personal involvement” factor). All the items presented factor loadings above 0.30 in their factor, and only three items presented cross-loadings in more than one factor. In these three cases the loadings on the main factor were above 0.50 and close to 0.30 in the secondary factor, indicating that the items were more related to the main factor (i.e., “personal involvement” factor in the first case, and “calling the cops” factor in the other two cases). The correlation between the “calling the cops” and the “personal involvement” factors was positive (*r* = 0.29), whereas the correlations between the “not my business” factor and the “calling the cops” and the “personal involvement” factors were negative (*r* = -0.55 and *r* = -0.28, respectively).

A CFA was conducted to test the extent of the effect of social desirability bias on the scale items. The CFA model posited the three previous content factors (i.e., “calling the cops,” “not my business,” “personal involvement”) and a new social desirability factor. The content factors were allowed to correlate with each other, whereas the social desirability factor was not correlated with any content factor. The WI-IPVAW items loaded on their main factor and also on the social desirability factor. The BIDR items were used as social desirability markers and only loaded on the social desirability factor. In addition, the BIDR items were constrained to have the same factor loadings on this factor. The model was estimated using WLSMV, converged normally, and showed an adequate fit (χ^2^ = 1130, *df* = 837; CFI = 0.93; TLI = 0.92; RMSEA = 0.049). The factor loadings are reported in **Table 2**.

**Table 2:** Confirmatory factor analysis with social desirability markers (Sample 1).

	Calling the cops	Not my business	Personal involvement	Social desirability
Item 1		0.63 (0.06)		-0.32 (0.08)
Item 2			0.63 (0.05)	0.20 (0.08)
Item 3			0.68 (0.05)	0.34 (0.09)
Item 4	0.70 (0.06)			0.01 (0.12)
Item 5	0.82 (0.06)			-0.21 (0.13)
Item 6			0.76 (0.04)	0.01 (0.08)
Item 7		0.68 (0.05)		-0.29 (0.08)
Item 8	0.69 (0.06)			-0.17 (0.10)
Item 9			0.77 (0.04)	0.20 (0.09)
Item 10	0.64 (0.05)			0.15 (0.09)
Item 11			0.78 (0.05)	0.33 (0.10)
Item 12			0.82 (0.04)	0.19 (0.09)
Item 13	0.79 (0.05)			-0.08 (0.11)
Item 14			0.53 (0.06)	0.24 (0.08)
Item 15		0.82 (0.04)		-0.15 (0.09)
Item 16		0.67 (0.05)		-0.25 (0.08)
Item 17	0.88 (0.06)			-0.28 (0.15)
Item 18	0.66 (0.06)			0.35 (0.08)
Item 19			0.73 (0.04)	0.28 (0.08)
Item 20			0.71 (0.05)	0.01 (0.08)
Item 21	0.81 (0.04)			-0.15 (0.11)
Item 22	0.66 (0.05)			0.31 (0.09)
Item 23			0.77 (0.04)	0.21 (0.09)
Item 24		0.61 (0.06)		-0.29 (0.08)
Item 25		0.68 (0.05)		-0.01 (0.09)
Item 26	0.81 (0.04)			-0.17 (0.11)
Item 27		0.81 (0.04)		-0.15 (0.09)
Item 28	0.55 (0.06)			0.32 (0.09)
Item 29	0.65 (0.07)			**0.41** (0.09)
Item 30	0.47 (0.07)			**0.38** (0.08)
Item 31	0.46 (0.07)			**0.47** (0.08)
BIDR1-8				0.37 (0.03)


**Each cell contains the factor loadings (SE in brackets). Empty cells indicate that the item does not load on that factor. BIDR 1-8: items from the impression management subscale of the Balanced Inventory of Desirable Response Short Form. Bold values: items that presented loadings in the social desirability factor higher than the social desirability markers (i.e., BIDR 1–8).**

Three items (e.g., “If a man insulted his partner in the street, I would say something to reprehend his action”; “If a man grabbed his partner’s arm aggressively in the street, forcing her to go with him, I would call the police”; “If a new couple in my building argued and yelled constantly, I would call the police”) presented factor loadings on the social desirability factor higher than the markers (λ = 0.37), and thus were removed from the scale. Ferrando (2005) recommends removing those items that present factor loadings above | 0.30| ; however, we decided to apply a more conservative criterion (i.e., removing only items that had factor loadings above the markers loading on the social desirability factor), since the internal consistency of the BIDR was moderate in the pilot study.

### Descriptive Analyses and Reliability

Sample 2 was used to assess the psychometric properties of the scale. Descriptive statistics and item-test corrected correlations can be found in **Table 3**. The descriptive statistics were in the same line as in the pilot study, with items slightly displaced to the right. The item means were around 4, with a standard deviation around 1, meaning that the respondents tended to endorse the upper intermediate categories (e.g., “somewhat likely,” “quite likely,” “very likely”). The skew statistics were moderate and negative for many of the items, and some of them also presented high kurtosis values, indicating that the items were not normally distributed. The item-test corrected correlations presented values above 0.40, indicating a strong relationship between the items and the total score of the scale. The overall internal consistency of the scale was again very good (Cronbach’s α = 0.94), and the internal consistency of each factor was also good (Cronbach’s α = 0.88, 0.84, and 0.92 for the “calling the cops,” “not my business,” and “personal involvement” factors, respectively).

**Table 3:** Descriptive statistics of the WI-IPVAW items (Sample 2).

	*M*	*SD*	Minimum	Maximum	Skew	Kurtosis	*SE*	*r*_item-test_
Item 1	3.07	1.31	1	6	0.28	-0.55	0.06	0.47
Item 2	4.10	1.40	1	6	-0.36	-0.78	0.06	0.63
Item 3	3.52	1.44	1	6	0.04	-0.97	0.06	0.65
Item 4	5.56	0.89	1	6	-2.54	7.18	0.04	0.46
Item 5	5.70	0.77	1	6	-3.48	14.31	0.03	0.43
Item 6	4.11	1.55	1	6	-0.49	-0.84	0.07	0.54
Item 7	2.87	1.35	1	6	0.31	-0.70	0.06	0.50
Item 8	5.38	1.05	1	6	-1.94	3.66	0.05	0.56
Item 9	3.77	1.42	1	6	-0.13	-0.81	0.06	0.71
Item 10	4.90	1.33	1	6	-1.21	0.70	0.06	0.60
Item 11	3.20	1.55	1	6	0.21	-1.02	0.07	0.66
Item 12	4.15	1.43	1	6	-0.39	-0.75	0.06	0.66
Item 13	5.55	0.90	1	6	-2.40	6.11	0.04	0.50
Item 14	3.14	1.60	1	6	0.26	-1.02	0.07	0.58
Item 15	2.24	1.25	1	6	0.94	0.25	0.06	0.53
Item 16	3.14	1.50	1	6	0.27	-0.95	0.07	0.55
Item 17	5.66	0.82	1	6	-3.03	10.26	0.04	0.43
Item 18	5.03	1.34	1	6	-1.41	1.20	0.06	0.57
Item 19	3.59	1.55	1	6	-0.03	-1.09	0.07	0.62
Item 20	4.43	1.48	1	6	-0.71	-0.49	0.07	0.60
Item 21	5.44	1.05	1	6	-2.21	4.68	0.05	0.44
Item 22	4.67	1.50	1	6	-0.99	-0.05	0.07	0.68
Item 23	3.71	1.47	1	6	-0.16	-0.91	0.07	0.68
Item 24	2.90	1.46	1	6	0.46	-0.74	0.07	0.50
Item 25	2.71	1.48	1	6	0.54	-0.74	0.07	0.41
Item 26	5.42	1.03	1	6	-2.21	5.17	0.05	0.51
Item 27	2.83	1.48	1	6	0.52	-0.68	0.07	0.50
Item 28	4.50	1.55	1	6	-0.78	-0.49	0.07	0.63


**M, mean; SD, standard deviation; Min, minimum; Max, maximum; SE, standard error for the Skew and Kurtosis statistics. r_*item-test*_, item-test corrected correlation.**

### Confirmatory Factor Analysis

Three models were estimated with Sample 2 to test the factor structure of the WI-IPVAW. The first model was a one-factor model in which all items loaded onto a general factor of “willingness to intervene in cases of IPVAW.” The second model was the three-factor model resulting from the pilot study, with three correlated factors differentiated by the responses to the scenarios described by the WI-IPVAW items (i.e., “calling the cops,” “not my business,” and “personal involvement”). The third model was a bifactor model with three specific factors reflecting different intervention preferences—as in the previous three-factor model—and a general, non-specific factor, of “willingness to intervene.” This general factor accounts for all the elements common to the specific factors. The specific factors account only for the core elements of their items, in this case the type of response to the scenarios described by the items. Thus, all the items loaded on their specific factor and also on the general factor. The factors were orthogonal, so they are not correlated. All models were estimated using WLSMV and the polychoric correlation matrix. All models converged normally.

The fit indices of the models are shown in **Table 4**. The one-factor model showed a poor fit to the data, presenting fit indices too far from their cut-offs. The three-factor model showed an acceptable RMSEA and a minimally acceptable CFI and TLI, which could be kept as the latent structure of the scale. However, adding a general dimension of “willingness to intervene” to the model substantially improved the fit of the model to the data. We therefore decided to retain the bifactor model.

**Table 4:** CFA fit indices (Sample 2).

	χ^2^	*df*	CFI	TLI	RMSEA
**Model**					
One-factor	3658.43	350	0.79	0.77	0.137 [0.133; 0.142]
Three-factor	1264.39	347	0.94	0.93	0.073 [0.068; 0.077]
Bifactor	1052.62	322	0.95	0.95	0.067 [0.063; 0.072]


**CFI, comparative fit index; TLI, Tucker-Lewis index; RMSEA, Root Mean Square Error of Approximation (95% CI in square brackets).**

The loadings of the bifactor model are displayed in **Table 5**. All the loadings for the specific factors were significant, with values above 0.30 in all the items except for items 2 and 3, whose loadings were around 0.20. The general factor loadings were all significant with values above |0.40|. Note that the “not my business” item loadings were negative in the general factor, reflecting that agreement with these items yielded a lower score on the general “willingness to intervene” factor. Overall, the general factor loadings were higher than in the specific factor. Furthermore, the percentage of common explained variance of the general “willingness to intervene” factor was 56.85%, whereas the specific “calling the cops” factor explained 23.16%, the “personal involvement” 11.04%, and the “not my business” 8.95% of the common explained variance.

**Table 5:** CFA item loadings on the bifactor model (Sample 2).

	Calling the cops	Not my business	Personal involvement	Willingness to intervene
Item 1		0.46 (0.04)		-0.48 (0.04)
Item 2			0.19 (0.05)	0.73 (0.03)
Item 3			0.20 (0.06)	0.75 (0.03)
Item 4	0.61 (0.04)			0.47 (0.05)
Item 5	0.65 (0.04)			0.52 (0.05)
Item 6			0.69 (0.04)	0.50 (0.05)
Item 7		0.41 (0.05)		-0.53 (0.04)
Item 8	0.59 (0.04)			0.57 (0.04)
Item 9			0.38 (0.05)	0.75 (0.03)
Item 10	0.49 (0.04)			0.58 (0.04)
Item 11			0.46 (0.04)	0.67 (0.03)
Item 12			0.41 (0.04)	0.70 (0.03)
Item 13	0.68 (0.04)			0.53 (0.05)
Item 14			0.46 (0.04)	0.57 (0.04)
Item 15		0.44 (0.05)		-0.62 (0.04)
Item 16		0.51 (0.04)		-0.56 (0.04)
Item 17	0.74 (0.04)			0.45 (0.05)
Item 18	0.48 (0.04)			0.56 (0.04)
Item 19			0.37 (0.05)	0.65 (0.04)
Item 20			0.60 (0.04)	0.59 (0.04)
Item 21	0.71 (0.03)			0.41 (0.05)
Item 22	0.47 (0.03)			0.67 (0.03)
Item 23			0.30 (0.05)	0.73 (0.03)
Item 24		0.53 (0.04)		-0.50 (0.04)
Item 25		0.44 (0.04)		-0.46 (0.04)
Item 26	0.65 (0.03)			0.50 (0.04)
Item 27		0.46 (0.05)		-0.55 (0.04)
Item 28	0.40 (0.03)			0.62 (0.03)


**Each cell contains the factor loadings (SE in brackets). Empty cells indicate that the item does not load on that factor*.*

### Measurement Invariance

Having retained the bifactor model as the latent structure of the scale, the measurement invariance of the scale was tested across genders using Sample 3. Item 5 was removed from these analyses since there were not enough responses in the lower categories for either the men’s or the women’s groups. A stepwise approach was used, testing first the configural invariance, and then comparing it with the metric, scalar, and strict invariance models. The fit indices of the models and the model comparisons are shown in **Tables 6**, **7**.

**Table 6:** Measurement invariance fit indices (Sample 3).

	χ^2^	*df*	CFI	TLI	RMSEA
Configural Model	1881.65	594	0.951	0.943	0.066 [0.063; 0.069]
Metric Invariance Model	1194.39	648	0.979	0.978	0.041 [0.037; 0.045]
Scalar Invariance Model	1410.71	776	0.976	0.978	0.040 [0.037; 0.044]
Partial Scalar Invariance Model	1355.42	766	0.978	0.980	0.039 [0.036; 0.043]
Strict Invariance Model	1658.43	739	0.965	0.967	0.050 [0.047; 0.053]


**CFI, comparative fit index; TLI, Tucker-Lewis index; RMSEA, Root Mean Square Error of Approximation (95% CI in square brackets).**

**Table 7:** Measurement invariance model comparisons (Sample 3).

	ΔCFI	ΔRMSEA	DIFFTEST	*df*	*p*
Configural Model					
Metric Invariance Model	-0.028	0.025	77.50	54	0.020
Scalar Invariance Model	0.003	0.001	280.69	128	0.000
Partial Scalar Invariance Model	-0.002	0.001	144.20	118	0.051
Strict Invariance Model	0.013	-0.110	61.87	30	0.001


**ΔCFI, change in CFI; ΔRMSEA, change in RMESEA; DIFFTEST, robust chi square difference testing; df, degree of freedom of the DIFTEST; p*, p-value of the DIFTEST.*

The configural model showed a good fit to the data, indicating that men and women conceptualize the latent construct in the same manner, and was used as a base line for the model comparisons. Then it was compared with the metric invariance model, which constrained the factor loadings to be equivalent across groups; we found that both CFI and RMSEA indices improved once the factor loadings were constrained. The DIFFTEST also showed that these improvements were marginally significant (*p* = 0.02). This is most likely due to the reduction in the number of parameters to estimate, making the model more parsimonious, and it is not an unusual phenomenon when conducting measurement invariance analysis with categorical data (e.g., Brummelman et al., 2015; Megías et al., 2017). Given the improvement in model fit and the reduction in the number of parameters to estimate, the metric invariance was supported.

The scalar invariance model, which besides the factor loading also constrained the item thresholds to be equal across gender, was compared with the metric model. Although the reduction in the CFI and RMSEA fit indices were between the cut-offs established by Cheung and Rensvold (2002), the DIFFTEST was significant (*p* < 0.001). The modification indices were then used to identify potential items to be unconstrained and test the partial scalar invariance model. The thresholds of two items (items 6 and 20) were allowed to vary across groups and we found that the partial invariance model did not differ from the metric model (*p* = 0.051). The partial scalar invariance model was thus supported.

Finally, the strict invariance model was tested, constraining the item variances to be equal across groups and comparing it with the partial invariance model. We found that the CFI decreased below the ΔCFI = 0.01 cut-off and the DIFFTEST was significant. Thus the strict invariance model could not be supported.

### Validity Analyses

Sample 3 was also used to conduct validity analyses. The correlations of the WI-IPVAW factorial scores with other related constructs are shown in **Table 8**. The general factor “willingness to intervene” was negatively related to acceptability of IPVAW, attitudes of victim blaming, and hostile sexism, implying that those respondents with higher scores on this factor tend to present lower levels of attitudes of acceptability, are less likely to blame victims of IPVAW, and show lower levels of sexist attitudes. On the other hand, the general factor was positively related with the perceived severity of IPVAW (those with higher scores on willingness to intervene tend to perceive IPVAW situations as more severe). Regarding the specific factors, the “calling the cops” factor showed a similar relation with these variables, although they were more moderate, whereas the “not my business” factor presented the opposite tendency: it was positively related with acceptability of IPVAW, attitudes of victim blaming, and hostile sexism, and negatively related to perceived severity of IPVAW. The “personal involvement” factor only presented a significant and negative relation to perceived severity.

**Table 8:** WI-IPVAW relationships with other variables (sample 3).

	Acceptability	Victim blaming	Perceived severity	Hostile sexism
Calling the cops	-0.13*	-0.21*	0.23*	-0.15*
Not my business	0.12*	0.11*	-0.11*	0.22*
Personal involvement	0.03	0.02	-0.12*	0.06
Willingness to intervene	-0.23*	-0.19*	0.25*	-0.20*


**^∗^p < 0.01*.*

A series of ANOVA were conducted with each factor to test differences across gender, age, and education level using the factor scores of the partial scalar invariance model. Regarding the general factor “willingness to intervene,” significant differences were found between genders, *F*(1) = 23.53, *p* < 0.001, η^2^ = 0.023, with a small effect size, women having higher values on this factor than men; marginal differences between age groups, *F*(3) = 3.09, *p* = 0.026, η^2^ = 0.009; and no differences for education level, *F*(3) = 1.30, *p* = 0.274, η^2^ = 0.004. The effect sizes of age and education levels were considered negligible, since they were below the 0.01 cut-off for small size effects (Miles and Shevlin, 2001).

Significant differences were also found in the specific “calling the cops” factor by gender, *F*(1) = 21.24, *p* < 0.001,η^2^ = 0.021, and age, *F*(3) = 3.73, *p* = 0.011, η^2^ = 0.011, both with a small effect size. Women scored higher on this factor than men, as did the respondents of the upper age categories (i.e., 35–54 and 55+) in comparison with the lower category (i.e., 18–24). Education level had no significant effect on this factor, *F*(3) = 0.89, *p* = 0.444, η^2^ = 0.002.

We found significant differences for the specific factor “not my business” by gender, *F*(1) = 5.45, *p* = 0.020, η^2^ = 0.005, although the effect size was considered negligible. No differences were found in this factor for age, *F*(3) = 2.27, *p* = 0.079, η^2^ = 0.006, or education level, *F*(3) = 2.27, *p* = 0.079, η^2^ = 0.002.

Regarding the specific factor “personal involvement,” significant differences were again found between genders, *F*(1) = 85.00, *p* < 0.001, η^2^ = 0.079, with a medium effect size, and age groups, *F*(3) = 5.08, *p* = 0.002, η^2^ = 0.015, with a small effect size. Men showed higher scores on this factor than women, and respondents in the upper age categories (i.e., 35–54 and 55+) presented higher scores than respondents in the lower age categories (i.e., 18–24 and 25–34). Again, education level had no effect on this factor, *F*(3) = 1.53, *p* = 0.197, η^2^ = 0.005.

### WI-IPVAW Shortened Forms

A combination of quantitative (i.e., social desirability loadings, bifactor model loadings, and whether items were invariant across genders) and qualitative criteria (i.e., the expert ratings) was used to decide which items should comprise the shortened versions of the scale (see **Table 9**). The items included were those that presented low loadings (i.e., below 0.20) on the social desirability factor used on the pilot study, with medium or high loadings (i.e., between 0.20–0.50, and above 0.50, respectively) on their specific and general factor, and that were invariant across genders. In addition to these criteria, the expert panel’s assessment of the representativeness and clarity of each item was also considered.

**Table 9:** Criteria for the shortened forms of the WI-IPVAW.

	Specific factor	SD factor loading	Specific factor loading	General factor loading	Invariant across gender	Expert ratings
Item 1	Not my business	Medium	Medium	Medium	Yes	CR
Item 2	Personal involvement	Low	Low	High	Yes	CR
Item 3	Personal involvement	Medium	Medium	High	Yes	CR
Item 4	Calling the cops	Low	High	Medium	Yes	
Item 5	Calling the cops	Low	High	High	Yes	R
Item 6	Personal involvement	Low	High	Medium	No	C
Item 7	Not my business	Low	Medium	High		CR
Item 8	Calling the cops	Low	High	High	Yes	CR
Item 9	Personal involvement	Low	Medium	High	Yes	CR
Item 10	Calling the cops	Low	Medium	High	Yes	CR
Item 11	Personal involvement	Medium	Medium	High	Yes	CR
Item 12	Personal involvement	Low	Medium	High	Yes	CR
Item 13	Calling the cops	Low	High	High	Yes	C
Item 14	Personal involvement	Low	Medium	High	Yes	R
Item 15	Not my business	Low	Medium	High	Yes	CR
Item 16	Not my business	Low	High	High	Yes	CR
Item 17	Calling the cops	Low	High	Medium	Yes	
Item 18	Calling the cops	Medium	Medium	High	Yes	CR
Item 19	Personal involvement	Low	Medium	High	Yes	C
Item 20	Personal involvement	Low	High	High	No	CR
Item 21	Calling the cops	Low	High	Medium	Yes	R
Item 22	Calling the cops	Medium	Medium	High	Yes	CR
Item 23	Personal involvement	Low	High	Medium	Yes	
Item 24	Not my business	Low			Yes	CR
Item 25	Not my business	Low	Medium	Medium	Yes	CR
Item 26	Calling the cops	Low	High	Medium	Yes	
Item 27	Not my business	Low	Medium	High	Yes	C
Item 28	Calling the cops	Medium	Medium	High	Yes	CR


**Expert ratings: items rated as clear (C) and/or representative (R) by the panel of experts*.*

#### Nine-Item Version of the WI-IPVAW Scale

To ensure content coverage, three items from each specific factor were selected to create a nine-item version of the WI-IPVAW scale (Smith et al., 2000), namely, items 2, 8, 9, 10, 12, 15, 16, 26, and 27. Although item 2 presented a low loading in the “calling the cops” factor, it was selected as it met the other criteria and the loading on the specific factor was close enough to the 0.20 cut-off for medium loadings (i.e., λ = 0.19). Sample 4 was then used to study the psychometric properties of the nine-item version of the scale. The internal consistency of this version was adequate (Cronbach’s α = 0.77), and the item-test corrected correlations were above 0.30 for all items except for item 26, for which it was 0.28. The factor structure of the nine-item version presented an excellent fit to the data when the bifactor model was fitted using WLSMV estimation with the polychoric correlation matrix (χ^2^(18) = 33.01, CFI = 0.98, TLI = 0.97, RMSEA = 0.065 [90% CI 0.027; 0.099]). Evidence for the validity based on its relationships with other constructs is reported with correlations in **Table 10**, which are in the same direction as for the complete WI-IPVAW version. Finally, the correlation between the nine-item version and the complete scale was very strong, *r* = 0.92, *t*(198) = 32.81, *p* < 0.001, suggesting that both versions provided similar assessments.

**Table 10:** WI-IPVAW short forms relationships with other variables (Sample 4).

	Acceptability	Victim blaming	Perceived severity	Hostile sexism
**Nine-item version**				
Calling the cops	-0.17*	-0.20*	0.33*	-0.10*
Not my business	0.10*	-0.07	0.05	0.13*
Personal involvement	-0.04	0.04	-0.04	0.02
Willingness to Intervene	-0.20*	-0.24*	0.29*	-0.16*
**Five-item version**				
Willingness to intervene	-0.23*	-0.29*	0.29*	-0.16*


**^∗^p < 0.01.**

#### Five-Item Version of the WI-IPVAW Scale

For circumstances in which space is very limited (e.g., large-scale surveys), a shorter version of the scale was created with a focus on the general factor. To this end, two items from the “calling the cops” and “personal involvement” factors and one item from the “not my business” factor were selected. These were the items that presented higher factor loadings on the general “willingness to intervene” factor in the nine-item version, namely, items 8, 9, 10, 12, and 27. Sample 4 was used to study the psychometric properties of this version of the scale. The internal consistency of the scale was again fair (Cronbach’s α = 0.73), and the item-test corrected correlations were above 0.30 for all items except for item 27 in this case, for which it was 0.27. A one-factor model was fitted to the five-item version of the scale since there were fewer than three items per specific factor, using WLSMV estimation. The model fitted reasonably well to the data (χ^2^(5) = 30.44, CFI = 0.96, TLI = 0.92, RMSEA = 0.150 [90% CI 0.099; 0.207]), although the residuals were below the 0.08 cut-off for a well-fitted model. The correlations between the “willingness to intervene” factor and the criterion-related variables were again in the same direction as for the complete version of the scale (see **Table 10**). The correlation between the five-item version and the complete version of the scale was high, *r* = 0.86, *t*(198) = 24, *p* < 0.001, although smaller than for the nine-item version.

## Discussion

In this paper, we described the development and psychometric properties of the long and short forms of the WI-IPVAW, a set of new self-report questionnaires assessing willingness to intervene in cases of IPVAW. Taken together, our results provide strong support for the reliability and validity of both the long and short versions of the WI-IPVAW scale.

Content validity of the WI-IPVAW was assessed during the scale development process using the ratings of a panel of experts, to ensure that the items adequately captured the different aspects of the construct. One of the advantages of the WI-IPVAW is that it also takes into account various community settings (next door house, streets, bars, etc.) where IPVAW can occur, as well as several expressions of this type of violence (e.g., verbal, threats, physical violence) in diverse situations and with different degrees of severity. The WI-IPVAW also includes a variety of potential responses to different IPVAW scenarios (e.g., talking to victims, personal involvement, calling the police, etc.). Tapping situation-specific responses across a range of settings provides greater ecological validity to this measure, and also facilitates future research on situational correlates of such attitudes (Carlo and Randall, 2002; Banyard, 2008; Banyard and Moynihan, 2011; Copp et al., 2016). Moreover, the effect of social desirability bias was controlled in a pilot study through a confirmatory factor analysis using social desirability markers (Ferrando, 2005, 2008). This analytical approach is one of the major strengths of the present study, because it allowed us to identify and remove items with higher loadings on the social desirability factor from the scale.

Regarding the internal structure of the scale, our results supported a bifactor model as the latent structure of the scale, as it presented the best fit to the data of all the models. In this model, each item loaded on one specific factor and also onto a general factor. This general factor (i.e., “willingness to intervene in cases of IPVAW”) captures the common variance of all items, reflecting the shared elements of the measured construct. On the other hand, the specific factors (i.e., “calling the cops,” “personal involvement,” and “not my business”) represent the remaining unique variance not attributable to the general factor. The model is orthogonal and thus the factors are uncorrelated, meaning that the general factor is assumed to be independent of the specific factors, and also that the specific factors are assumed to be different from and independent of each other (e.g., Chen et al., 2006; Gibbins et al., 2012). In addition, our results highlight the relevance of the general factor since most of the loadings presented higher values on the general factor than on their respective specific factor. The general factor also accounted for the largest proportion of the common explained variance, 56.85%. The “calling the cops” factor accounted for almost half of the remaining common variance, 23.16%, whereas the “personal involvement” and “not my business” specific factors explained the rest, 11.04 and 8.95%, respectively.

We also conducted measurement invariance analyses of the WI-IPVAW across genders. A partial scalar invariance model was supported, showing that men and women conceptualize the underlying latent structure in the same manner (configural invariance), that the scale unit is the same, and thus the items are interpreted similarly by men and women (metric invariance), and that the thresholds of the items are the same for both genders, as the factorial scores were comparable across gender groups (scalar invariance). However, the threshold parameters of two items (items 6 and 21) were allowed to vary across groups, implying that men and women do not share the same distribution on these items. To obtain comparable scores for men and women in the general “willingness to intervene” factor and in the specific factors, researchers and practitioners could remove items 6 and 21 from the scale. We recommend, however, using the invariant items as anchor items and treating these two items differently for each gender. To this end, we provide an Mplus syntax to compute this model in Appendix 2 (see Supplementary Material).

Regarding validity analyses based on the relationships of the WI-IPVAW with other variables, we found that the general factor (i.e., “willingness to intervene in cases of IPVAW”) was significantly associated with a set of relevant variables linked to IPVAW. Thus, as expected, respondents with higher scores on the WI-IPVAW (i.e., those more willing to intervene), perceive IPVAW situations as more severe, find IPVAW less acceptable, have fewer victim-blaming attitudes, and score lower in hostile sexism. This supports the idea that willingness to intervene in cases of IPVAW reflects the personal level of tolerance and acceptance of this type of violence and suggests that attitudes toward intervention in cases of IPVAW are also linked to attitudes justifying IPVAW, such as victim blaming, and to hostility toward women (Glick et al., 2002; Taylor and Sorenson, 2005; Gracia et al., 2014; Herrero et al., 2017; Ivert et al., 2018). With respect to the specific factors, both “calling the cops” and “not my business,” were related as expected (i.e., the first positively and the second negatively) with the same set of variables. For example, those scoring high in the “not my business” factor tended to perceive IPVAW as less severe and more acceptable and scored higher in both victim-blaming attitudes and hostile sexism. Interestingly, the “personal involvement” factor was related, negatively, only with the perceived severity of IPVAW, suggesting that the more severe an IPVAW situation is perceived, the more other intervention preferences are favored, as greater personal costs or negative consequences may be involved. For example, as Gracia et al. (2009) observed, reporting incidents of IPVAW to the police is more likely among those who tend to perceive these incidents as more severe.

In this study, we also developed two shortened versions of the WI-IPVAW scale. The full WI-IPVAW scale is a relatively lengthy questionnaire. The length of questionnaires often prevents their inclusion in population surveys where space is limited and expensive, or in studies where time is an issue. Large-scale surveys tend to resort to single items addressing these attitudes or use a set of questions with unknown reliability or validity (Richins, 2004; Gracia and Lila, 2015). On the other hand, shortened versions can have the drawback of limited reliability and validity, which makes it particularly important to ensure that short versions of questionnaires retain their psychometric soundness (Smith et al., 2000; Stanton et al., 2002; Kovacs et al., 2017). As Smith et al. (2000) point out, rigorous application of psychometric principles is crucial when validating short forms. In the present study, two short nine- and six-item versions of the parent WI-IPVAW scale were constructed based on quantitative and qualitative criteria (Goetz et al., 2013), supporting the adequate transfer of validity from the parent form of the WI-IPVAW to the two short forms. The complete and short versions of the WI-IPVAW demonstrated high reliability as well as construct validity as they were strongly related to acceptability of IPVAW, victim-blaming attitudes, perceived severity of IPVAW, and hostile sexism. Although some loss of reliability is inevitable, our results provide strong empirical support for the high quality of their psychometric properties of the short versions of the WI-IPVAW scale. When research or survey needs (large-scale surveys, limited space or time, etc.) require the use of short forms, our results demonstrate that both the nine- and the five-item short forms are reliable and valid alternatives to the most comprehensive and broader assessment of willingness to intervene in cases of IPVAW provided by the long version of the WI-IPVAW (both reduced versions presented a high correlation with the parent WI-IPVAW scale). For example, the nine-item WI-IPVAW short scale showed not only adequate reliability, but also allowed meaningful assessment of both the general non-specific factor expressing the willingness to intervene in cases of IPVAW, and the three specific factors reflecting different intervention preferences (adequate representation of the construct is ensured by incorporating three items from each of the specific factors of the original scale). In turn, the five-item WI-IPVAW short scale is particularly recommended for the reliable and valid assessment of the general “willingness to intervene” factor when space and/or time constraints are an issue, but this construct is still important for research or policy-making purposes. The five-item version only mapped the general factor as there were not enough items to preserve the original latent structure of the scale. The scores on the general factor of the five-item version presented a similar pattern when related to acceptability of IPVAW, attitudes of victim blaming, perceived severity, and hostile sexism.

This study is not without limitations. Although social desirability was controlled in the pilot study following the procedure proposed by Ferrando (2005), the items used as social desirability markers presented a mediocre reliability, and thus these results should be taken with caution. Regarding the measurement invariance, although the partial scalar invariance level for the WI-IPVAW across genders was supported, further research is needed to establish whether this instrument is also invariant across age and education level groups. The online sampling method is another limitation of the study, as it has some tradeoffs that limit its generalizability. Although this sampling strategy is effective for obtaining large sample sizes in a short period of time and is also cost-effective, it is more difficult to verify the socio-demographical information provided by the participants (Thornton et al., 2016; Topolovec-Vranic and Natarajan, 2016). Self-selection bias is another issue, since the respondents who agreed to participate might also be those that are more motivated. In addition, it is important to note that the WI-IPVAW was developed in the Spanish socio-cultural context. Spain is among the countries with the lowest IPVAW lifetime prevalence in the EU (Vives-Cases et al., 2011; European Union Agency for Fundamental Rights, 2014; Gracia and Merlo, 2016). This is particularly interesting given that other European countries have considerably higher levels of gender equality than Spain (Gracia and Merlo, 2016). As to whether these differences in prevalence are linked to differences across countries regarding public attitudes such as willingness to intervene in cases of IPVAW, future research is needed to adapt and validate the WI-IPVAW scale to other cultural settings (Gracia and Lila, 2015; Boira et al., 2016).

The study also has practical implications. Addressing attitudes towards IPVAW, such as willingness to intervene in cases of IPVAW, and advancing in their conceptualization, measurement, prevalence, and determinants is central to monitoring social changes in such attitudes and to better informing prevention and intervention strategies (Powell and Webster, 2018). Public willingness to intervene in cases of IPVAW reflects the level of tolerance and acceptability of IPVAW, and when these attitudes are held collectively at different levels of aggregation (e.g., social groups, neighborhoods, communities, countries), they are able to create a social climate that can help to legitimize or deter this type of violence (Browning, 2002; Emery et al., 2011; Heise, 2011; Wright and Benson, 2011; Heise and Kotsadam, 2015; Voith, 2017; Marco et al., 2018). For example, a public education strategy should consider targeting those social groups or communities were IPVAW risk is higher, and these attitudes can be more commonly held (Gracia and Tomás, 2014; Gracia et al., 2015a). In this regard, the different versions of the WI-IPVAW—especially the short versions, which are more appropriate for survey type research—can be used to assess pro- or non-intervention norms at different aggregation levels, such as neighborhoods or communities, when they are considered as key targets for social and community intervention strategies addressing the prevalence of IPVAW and its correlates, such as public attitudes (Gracia, 2014; Gracia et al., 2015a; Voith, 2017). As Klein et al. (1997, p. 90) state, “we need to educate people to recognize that they have a role in helping battered women and to teach them that their behavior matters, and showed them how to get involved.” In this regard, and in line with Gracia et al. (2009), public education efforts must promote attitudes that reinforce the helping role of the victim’s social circle in order to increase feelings of social and personal responsibility about the high prevalence of IPVAW in our societies. Increasing the likelihood of public intervention to help IPVAW victims, not only among the general public but also within professional groups (social services, health, law enforcement, etc.), can contribute to deter and reduce this major social and public health problem (Gracia et al., 2014; Ferrer-Perez et al., 2016; López-Ossorio et al., 2016; Touza-Garma, 2017). The WI-IPVAW therefore offers a useful instrument to assess prevention policies and public education campaigns aiming to promote a more responsive social environment in cases of IPVAW.

## Ethics Statement

This study was performed in accordance with the Declaration of Helsinki. Informed consent information was supplied and implied through participation in the on-line survey. The study and protocol were reviewed and approved by the University of Valencia Ethics Committee.

## Author Contributions

EG conceived the study and supervised the writing of the manuscript. MM-F designed the analytic strategy, contributed to developing materials, conducted the statistical analysis, and wrote the methods and results sections of the manuscript. MM contributed to developing materials, data collection, and writing some parts of the manuscript. FS contributed to developing materials, data collection, and writing some parts of the manuscript. VV contributed to developing materials, data collection, and writing some parts of the manuscript. ML coordinated the data collection and contributed to the writing of the manuscript.

## Conflict of Interest Statement

The authors declare that the research was conducted in the absence of any commercial or financial relationships that could be construed as a potential conflict of interest.

## References

[B1] Ansara D. L. Hindin M. J. (2010). Formal and informal help-seeking associated with women’s and men’s experience of intimate partner violence in Canada. *Soc. Sci. Med.* 70 1011–1018. 10.1016/j.socscimed.2009.12.009 20122774

[B2] Asparouhov T. Muthén B. (2010). *Weighted Least Squares Estimation With Missing Data.* Available at: http://www.statmodel.com/download/GstrucMissingRevision.pdf

[B3] Asparouhov T. Muthén B. Muthén B. O. (2006). Robust chi square difference testing with mean and variance adjusted test statistics. *Matrix* 1 1–6. 10.1177/0013164416683754 29795956 PMC5965660

[B4] Baldry A. C. Pacilli M. G. Pagliaro S. (2015). She’s not a person.. she’s just a woman! Infra-Humanization and intimate partner violence. *J. Interpers. Violence* 30 1567–1582. 10.1177/0886260514540801 24986191

[B5] Baldry A. C. Pagliaro S. (2014). Helping victims of intimate partner violence: the influence of group norms among lay people and the police. *Psychol. Violence* 4 334–347. 10.1037/a0034844

[B6] Banyard V. Moynihan M. M. (2011). Variation in bystander behavior related to sexual and intimate partner violence prevention: correlates in a sample of college students. *Psychol. Violence* 1 287–301. 10.1037/a0023544

[B7] Banyard V. L. (2008). Measurement and correlates of prosocial bystander behavior: the case of interpersonal violence. *Violence Vict.* 23 83–97. 10.1891/0886-6708.23.1.83 18396583

[B8] Banyard V. L. Moynihan M. M. Cares A. C. Warner R. (2014). How do we know if it works? Measuring outcomes in bystander-focused abuse prevention on campuses. *Psychol. Violence* 4 101–115. 10.1037/a0033470

[B9] Beck C. T. Gable R. K. (2001). Ensuring content validity: illustration of the process. *J. Nurs. Meas.* 9 201–215. 11696942

[B10] Bentler P. M. (1995). *EQS Program Manual.* Encino, CA: Multivariate Software Inc.

[B11] Boira S. Carbajosa P. Méndez R. (2016). Miedo, conformidad y silencio: la violencia en las relaciones de pareja en áreas rurales de Ecuador [Fear, conformity and silence. Intimate partner violence in rural areas of Ecuador]. *Psychosoc. Interv.* 25 9–17. 10.1016/j.psi.2015.07.008

[B12] Branch K. A. Richards T. N. Dretsch E. C. (2013). An exploratory analysis of college students’ response and reporting behavior regarding intimate partner violence victimization and perpetration among their friends. *J. Interpers. Violence* 28 3386–3399. 10.1177/0886260513504494 24114762

[B13] Browning C. R. (2002). The span of collective efficacy: extending social disorganization theory to partner violence. *J. Marriage Fam.* 64 833–850. 10.1111/j.1741-3737.2002.00833.x

[B14] Brummelman E. Thomaes S. Nelemans S. A. Orobio de Castro B. Bushman B. J. (2015). My child is God’s gift to humanity: development and validation of the parental overvaluation scale (POS). *J. Pers. Soc. Psychol.* 108 665–679. 10.1037/pspp0000012 25365035

[B15] Campbell J. C. (2002). Health consequences of intimate partner violence. *Lancet* 359 1331–1336. 10.1016/S0140-6736(02)08336-8 11965295

[B16] Carlo G. Randall B. A. (2002). The development of a measure of prosocial behaviors for late adolescents. *J. Youth Adolesc.* 31 31–44. 10.1023/A:1014033032440

[B17] Carlson B. E. Worden A. P. (2005). Attitudes and beliefs about domestic violence: results of a public opinion survey: I. Definitions of domestic violence, criminal domestic violence, and prevalence. *J. Interpers. Violence* 20 1197–1218. 10.1177/0886260505278530 16162486

[B18] Chen F. F. West S. G. Sousa K. H. (2006). A comparison of bifactor and second-order models of quality of life. *Multivariate Behav. Res.* 41 189–225. 10.1207/s15327906mbr4102_;5 26782910

[B19] Cheung G. W. Rensvold R. B. (2002). Evaluating goodness-of-fit indexes for testing measurement invariance. *Struct. Equat. Model.* 9 233–255. 10.1097/NNR.0b013e3182544750 22551991 PMC3361901

[B20] Cinquegrana V. Baldry A. C. Pagliaro S. (2018). Intimate partner violence and bystanders’ helping behaviour: an experimental study. *J. Aggress. Conflict Peace Res.* 10 24–35.

[B21] Consejo General del Poder Judicial (2016). *La Violencia Sobre la Mujer en la Estadística Judicial: Anual 2016 [Violence Against Women in Judicial Statistics: YEAR 2016].* Available at: http://www.poderjudicial.es/cgpj/es/Temas/Violencia-domestica-y-de-genero/Actividad-del-Observatorio/Datos-estadisticos/La-violencia-sobre-la-mujer-en-la-estadistica-judicial--Anual-2016

[B22] Copp J. E. Giordano P. C. Longmore M. A. Manning W. D. (2016). The development of attitudes toward intimate partner violence: an examination of key correlates among a sample of young adults. *J. Interpers. Violence.* 10.1177/0886260516651311 [Epub ahead of print]. 27229921 PMC5123960

[B23] Delgado-Rico E. Carretero-Dios H. Ruch W. (2012). Content validity evidences in test development: an applied perspective. *Int. J. Clin. Health Psychol.* 12 449–459. 10.1111/jan.12741 26265326

[B24] Devries K. M. Mak J. Y. García-Moreno C. Petzold M. Child J. C. Falder G. (2013). The global prevalence of intimate partner violence against women. *Science* 340 1527–1528. 10.1126/science.1240937 23788730

[B25] Devries K. M. Watts C. Yoshihama M. Kiss L. Schraiber L. B. Deyessa N. (2011). Violence against women is strongly associated with suicide attempts: evidence from the WHO multi-country study on women’s health and domestic violence against women. *Soc. Sci. Med.* 73 79–86. 10.1016/j.socscimed.2011.05.006 21676510

[B26] Ellsberg M. Jansen H. A. Heise L. Watts C. H. Garcia-Moreno C. (2008). Intimate partner violence and women’s physical and mental health in the WHO multi-country study on women’s health and domestic violence: an observational study. *Lancet* 371 1165–1172. 10.1016/S0140-6736(08)60522-X 18395577

[B27] Emery C. R. Jolley J. M. Wu S. (2011). Desistance from intimate partner violence: the role of legal cynicism, collective efficacy, and social disorganization in Chicago neighborhoods. *Am. J. Community Psychol.* 48 373–383. 10.1007/s10464-010-9362-5 20963479

[B28] European Commission (2016). *Gender Based Violence (Special Eurobaromenter 449 – Wave EB85.3 – TNS Opinion & Social).* Brussels: European Commission.

[B29] European Union Agency for Fundamental Rights (2014). *Violence Against Women: An EU-Wide Survey.* Luxembourg: Publications Office of the European Union.

[B30] Expósito F. Moya M. C. Glick P. (1998). Sexismo ambivalente: medición y correlatos [Ambivalent sexism: measurement and correlates]. *Rev. Psicol. Soc.* 13 159–169. 10.1174/021347498760350641

[B31] Ferrando P. J. (2005). Factor analytic procedures for assessing social desirability in binary items. *Multivariate Behav. Res.* 40 331–349. 10.1207/s15327906mbr4003_;3 26794687

[B32] Ferrando P. J. (2008). The impact of social desirability bias on the EPQ-R item scores: an item response theory analysis. *Pers. Individ. Diff.* 44 1784–1794. 10.1016/j.paid.2008.02.005

[B33] Ferrer-Perez V. A. Ferreiro-Basurto V. Navarro-Guzmán C. Bosch-Fiol E. (2016). Programas de intervención con maltratadores en España: la perspectiva de los/as profesionales [Batterer intervention programs in Spain: the professionals’ perspective]. *Psychosoc. Interv.* 25 159–168. 10.1016/j.psi.2016.06.001

[B34] Fincham F. D. Cui M. Braithwaite S. Pasley K. (2008). Attitudes toward intimate partner violence in dating relationships. *Psychol. Assess.* 20 260–269. 10.1037/1040-3590.20.3.260 18778162

[B35] Flood M. Pease B. (2009). Factors influencing attitudes to violence against women. *Trauma Violence Abuse* 10 125–142. 10.1177/1524838009334131 19383630

[B36] Garcia-Moreno C. Jansen H. A. Ellsberg M. Heise L. Watts C. H. (2006). Prevalence of intimate partner violence: findings from the WHO multi-country study on women’s health and domestic violence. *Lancet* 368 1260–1269. 10.1016/S0140-6736(06)69523-8 17027732

[B37] Gibbins C. Toplak M. E. Flora D. B. Weiss M. D. Tannock R. (2012). Evidence for a general factor model of ADHD in adults. *J. Atten. Disord.* 16 635–644. 10.1177/1087054711416310 22076604

[B38] Glick P. Fiske S. T. (1996). The ambivalent sexism inventory: differentiating hostile and benevolent sexism. *J. Pers. Soc. Psychol.* 70:491 10.1037/0022-3514.70.3.491

[B39] Glick P. Fiske S. T. Mladinic A. Saiz J. L. Abrams D. Masser B. (2000). Beyond prejudice as simple antipathy: hostile and benevolent sexism across cultures. *J. Pers. Soc. Psychol.* 79 763–775. 10.1037/0022-3514.79.5.763 11079240

[B40] Glick P. Sakalli–Ugurlu N. Ferreira M. C. Aguiar de Souza M. (2002). Ambivalent sexism and attitudes toward wife abuse in Turkey and Brazil. *Psychol. Women Q.* 26 292–297. 10.1111/1471-6402.t01-1-00068

[B41] Goetz C. Coste J. Lemetayer F. Rat A. C. Montel S. Recchia S. (2013). Item reduction based on rigorous methodological guidelines is necessary to maintain validity when shortening composite measurement scales. *J. Clin. Epidemiol.* 66 710–718. 10.1016/j.jclinepi.2012.12.015 23566375

[B42] Gracia E. (2004). Unreported cases of domestic violence against women: towards an epidemiology of social silence, tolerance, and inhibition. *J. Epidemiol. Community Health* 58 536–537. 10.1136/jech.2003.019604 15194711 PMC1732820

[B43] Gracia E. (2014). Intimate partner violence against women and victim-blaming among Europeans. *Bull. World Health Organ.* 92 380–381. 10.2471/BLT.13.131391 24839328 PMC4007130

[B44] Gracia E. García F. Lila M. (2009). Public responses to intimate partner violence against women: the influence of perceived severity and personal responsibility. *Span. J. Psychol.* 12 648–656. 10.1017/S1138741600002018 19899665

[B45] Gracia E. García F. Lila M. (2011). Police attitudes toward policing partner violence against women: do they correspond to different psychosocial profiles? *J. Interpers. Violence* 26 189–207. 10.1177/0886260510362892 20448237

[B46] Gracia E. García F. Lila M. (2014). Male police officers’ law enforcement preferences in cases of intimate partner violence versus non-intimate interpersonal violence: do sexist attitudes and empathy matter? *Crim. Justice Behav.* 41 1195–1213. 10.1177/0093854814541655

[B47] Gracia E. Herrero J. (2006). Public attitudes toward reporting partner violence against women and reporting behavior. *J. Marriage Fam.* 68 759–768. 10.1111/j.1741-3737.2006.00288.x

[B48] Gracia E. Lila M. (2015). *Attitudes Towards Violence Against Women in the EU.* Luxembourg: Publication Office of the European Union.

[B49] Gracia E. López-Quílez A. Marco M. Lladosa S. Lila M. (2015a). The spatial epidemiology of intimate partner violence: do neighborhoods matter? *Am. J. Epidemiol.* 182 58–66. 10.1093/aje/kwv016 25980418

[B50] Gracia E. Merlo J. (2016). Intimate partner violence against women and the Nordic paradox. *Soc. Sci. Med.* 157 27–30. 10.1016/j.socscimed.2016.03.040 27058634

[B51] Gracia E. Rodriguez C. M. Lila M. (2015b). Preliminary evaluation of an analog procedure to assess acceptability of intimate partner violence against women: the partner violence acceptability movie task. *Front. Psychol.* 6:1567. 10.3389/fpsyg.2015.01567 26528220 PMC4600898

[B52] Gracia E. Tomás J. M. (2014). Correlates of victim-blaming attitudes regarding partner violence against women among the Spanish general population. *Violence Against Women* 20 26–41. 10.1177/1077801213520577 24476756

[B53] Grimm P. (2010). “Social desirability bias,” in *Wiley International Encyclopedia of Marketing*, eds Sheth J. Malhotra N. K. (New York, NY: John Wiley & Sons).

[B54] Guedes A. Bott S. Garcia-Moreno C. Colombini M. (2016). Bridging the gaps: a global review of intersections of violence against women and violence against children. *Global Health Action* 9:31516. 10.3402/gha.v9.31516 27329936 PMC4916258

[B55] Hamby S. Weber M. C. Grych J. Banyard V. (2015). What difference do bystanders make? The association of bystander involvement with victim out-comes in a community sample. *Psychol. Violence* 6 91–102. 10.1037/a0039073

[B56] Hart C. M. Ritchie T. D. Hepper E. G. Gebauer J. E. (2015). The balanced inventory of desirable responding short form (BIDR-16). *Sage Open* 5 1–9. 10.1177/2158244015621113

[B57] Heise L. (1998). Violence against women: an integrated, ecological framework. *Violence Against Women* 4 262–290. 10.1177/1077801298004003002 12296014

[B58] Heise L. (2011). *What Works to Prevent Partner Violence? An Evidence Overview.* London: STRIVE.

[B59] Heise L. L. Kotsadam A. (2015). Cross-national and multilevel correlates of partner violence: an analysis of data from population-based surveys. *Lancet Glob. Health* 3 e332–e340. 10.1016/S2214-109X(15)00013-3 26001577

[B60] Herrero J. Rodríguez F. J. Torres A. (2017). Acceptability of partner violence in 51 societies: the role of sexism and attitudes toward violence in social relationships. *Violence Against Women* 23 351–367. 10.1177/1077801216642870 27126860

[B61] Hu L. T. Bentler P. M. (1999). Cutoff criteria for fit indices in covariance structure analysis: conventional criteria versus new alternatives. *Struct.l Equat. Model.* 6 1–55. 10.1080/10705519909540118

[B62] Ivert A. K. Merlo J. Gracia E. (2018). Country of residence, gender equality and victim blaming attitudes about partner violence: a multilevel analysis in EU. *Eur. J. Public Health.* 28 559–564. 10.1093/eurpub/ckx138 29036678

[B63] Jewkes R. Flood M. Lang J. (2015). From work with men and boys to changes of social norms and reduction of inequities in gender relations: a conceptual shift in prevention of violence against women and girls. *Lancet* 385 1580–1589. 10.1016/S0140-6736(14)61683-4 25467578

[B64] Klein E. Campbell J. Soler E. Ghez M. (1997). *Ending Domestic Violence: Changing Public Perceptions/Halting the Epidemic.* Thousand Oaks, CA: Sage.

[B65] Koepsell J. K. Kernic M. A. Holt V. L. (2006). Factors that influence battered women to leave their abusive relationships. *Violence Vict.* 21 131–147. 10.1891/vivi.21.2.131 16642735

[B66] Kovacs C. Batinic B. Stiglbauer B. Gnambs T. (2017). Development of a shortened version of the latent and manifest benefits of work (LAMB) scale. *Eur. J. Psychol. Assess.* 10.1027/1015-5759/a000434

[B67] Latané B. Darley J. M. (1970). *The Unresponsive Bystander: Why Doesn’t He Help?* New York, NY: Appleton-Century Crofts.

[B68] Liang B. Goodman L. Tummala-Narra P. Weintraub S. (2005). A theoretical framework for understanding help-seeking processes among survivors of intimate partner violence. *Am. J. Commun. Psychol.* 36 71–84. 10.1007/s10464-005-6233-6 16134045

[B69] Lila M. Gracia E. García F. (2013). Ambivalent sexism, empathy and law enforcement attitudes towards partner violence against women among male police officers. *Psychol. Crime Law* 19 907–919. 10.1080/1068316X.2012.719619

[B70] Lila M. Oliver A. Catalá-Miñana A. Galiana L. Gracia E. (2014). The intimate partner violence responsibility attribution scale (IPVRAS). *Eur. J. Psychol. Appl. Legal Context* 6 29–36. 10.5093/ejpalc2014a4

[B71] López-Ossorio J. J. González-Álvarez J. L. Andrés-Pueyo A. (2016). Eficacia predictiva de la valoración policial del riesgo de la violencia de género [Predictive effectiveness of the police risk assessment in intimate partner violence]. *Psychosoc. Interv.* 25 1–7. 10.1016/j.psi.2015.10.002

[B72] MacCallum R. C. Browne M. W. Sugawara H. M. (1996). Power analysis and determination of sample size for covariance structure modeling. *Psychol. Methods* 1 130–149. 10.1037/1082-989X.1.2.130

[B73] Marco M. Gracia E. López-Quílez A. (2018). The university campus environment as a protective factor for intimate partner violence against women: an exploratory study. *J. Commun. Psychol.* 10.1002/jcop.21980 30565738

[B74] Martín-Fernández M. Gracia E. Lila M. (2018b). Assessing victim-blaming attitudes in cases of intimate partner violence against women: Development and validation of the VB-IPVAW scale. *Psychosoc. Interv.* 10.5093/pi2018a18

[B75] Martín-Fernández M. Gracia E. Marco M. Vargas V. Santirso F. A. Lila M. (2018a). Measuring acceptability of intimate partner violence against women: development and validation of the A-IPVAW scale. *Eur. J. Psychol. Appl. Legal Context* 10 26–34. 10.5093/ejpalc2018a3

[B76] McCart M. R. Smith D. Sawyer G. K. (2010). Help seeking among victims of crime: a review of the empirical literature. *J. Trauma Stress* 23 198–206. 10.1002/jts.20509 20336674 PMC3803158

[B77] McDonnell K. A. Burke J. G. Gielen A. C. O’Campo P. Weidl M. (2011). Women’s perceptions of their community’s social norms towards assisting women who have experienced intimate partner violence. *J. Urban Health* 88 240–253. 10.1007/s11524-011-9546-9 21336504 PMC3079036

[B78] McMahon S. Allen C. T. Postmus J. L. McMahon S. M. Peterson N. A. Hoffman M. L. (2014). Measuring bystander attitudes and behavior to prevent sexual violence. *J. Am. Coll. Health* 62 58–66. 10.1080/07448481.2013.849258 24313697

[B79] Megías J. L. Toro-García V. Carretero-Dios H. (2017). The acceptance of myths about intimate partner violence against women (AMIVAW) scale: development and validation in Spanish and English. *Psychol. Women Q.* 42 44–61. 10.1177/0361684317742638

[B80] Miles J. Shevlin M. (2001). *Applying Regression and Correlation: A Guide for Students and Researchers.* London: Sage.

[B81] Milfont T. L. Fischer R. (2010). Testing measurement invariance across groups: applications in crosscultural research. *Int. J. Psychol. Res.* 3 111–121. 10.21500/20112084.857

[B82] Ministerio de Sanidad Servicios Sociales e Igualdad (2015). *Macroencuesta de Violencia Contra la Mujer 2015 [Survey on Violence Against Women 2015].* Madrid: Ministerio de Sanidad, Servicios Sociales e Igualdad.

[B83] Muthén B. Kaplan D. (1985). A comparison of some methodologies for the factor analysis of non-normal Likert variables. *Br. J. Math. Stat. Psychol.* 38 171–189. 10.1111/j.2044-8317.1985.tb00832.x

[B84] Muthén B. Kaplan D. (1992). A comparison of some methodologies for the factor analysis of non-normal Likert variables: a note on the size of the model. *Br. J. Math. Stat. Psychol.* 45 19–30. 10.1111/j.2044-8317.1992.tb00975.x

[B85] Muthén L. K. Muthén B. O. (2010). *Mplus User’s Guide.* Los Angeles, CA: Author.

[B86] Powell A. Webster K. (2018). Cultures of gendered violence: an integrative review of measures of attitudinal support for violence against women. *Austral. N. Z. J. Criminol.* 51 40–57. 10.1177/0004865816675669

[B87] R Core Team (2017). *R: A Language and Environment for Statistical Computing.* Vienna: R Foundation for Statistical Computing.

[B88] Revelle W. (2016). *psych: Procedures for Personality and Psychological Research.* Evanston, IL: Northwestern University.

[B89] Richins M. L. (2004). The material values scale: measurement properties and development of a short form. *J. Consum. Res.* 31 209–219. 10.1086/383436

[B90] Rizo C. F. Macy R. J. (2011). Help seeking and barriers of Hispanic partner systematic review of the literature. *Aggress. Violent Behav.* 6 250–264. 10.1016/j.avb.2011.03.004 20602188

[B91] Rollero C. Glick P. Tartaglia S. (2014). Psychometric properties of short versions of the ambivalent sexism inventory and ambivalence toward men inventory. *TPM. Test. Psychometr. Methodol. Appl. Psychol.* 21 149–159.

[B92] Smith G. T. McCarthy D. M. Anderson K. G. (2000). On the sins of short-form development. *Psychol. Assess.* 12 102–111. 10.1037/1040-3590.12.1.102 10752369

[B93] Stanton J. M. Sinar E. F. Balzer W. K. Smith P. C. (2002). Issues and strategies for reducing the length of self-report scales. *Pers. Psychol.* 55 167–194. 10.1111/j.1744-6570.2002.tb00108.x

[B94] Stein J. L. (2007). Peer educators and close friends as predictors of male college students’ willingness to prevent rape. *J. College Stud. Dev.* 48 75–89. 10.1353/csd.2007.0008

[B95] Stöckl H. Devries K. Rotstein A. Abrahams N. Campbell J. Watts C. (2013). The global prevalence of intimate partner homicide: a systematic review. *Lancet* 382 859–865. 10.1016/S0140-6736(13)61030-2 23791474

[B96] Taylor C. A. Sorenson S. B. (2005). Community-based norms about intimate attributions of fault and responsibility into context. *Sex Roles* 53 573–589. 10.1007/s11199-006-9155-3

[B97] Taylor E. Banyard V. Grych J. Hamby S. (2016). Not all behind closed doors: examining bystander involvement in intimate partner violence. *J. Interpers. Violence.* 10.1177/0886260516673629 [Epub ahead of print]. 29294613

[B98] Thornton L. Batterham P. J. Fassnacht D. B. Kay-Lambkin F. Calear A. L. Hunt S. (2016). Recruiting for health, medical or psychosocial research using Facebook: systematic review. *Internet Intervent.* 4 72–81. 10.1016/j.invent.2016.02.001 PMC6096238 30135792

[B99] Timmerman M. E. Lorenzo-Seva U. (2011). Dimensionality assessment of ordered polytomous items with parallel analysis. *Psychol. Methods* 16 209–220. 10.1037/a0023353 21500916

[B100] Topolovec-Vranic J. Natarajan K. (2016). The use of social media in recruitment for medical research studies: a scoping review. *J. Med. Internet Res.* 18 e286. 10.2196/jmir.5698 27821383 PMC5118584

[B101] Touza-Garma C. (2017). Influence of health personnel’s attitudes and knowledge in the detection and reporting of elder abuse: an exploratory systematic review. *Psychosoc. Intervent.* 26 73–91. 10.1016/j.psi.2016.11.001

[B102] Vargas V. Lila M. Catalá-Miñana A. (2015). >Influyen las diferencias culturales en los resultados de los programas de intervención con maltratadores?: un estudio con agresores españoles y latinoamericanos [Do cultural differences influence batterer intervention program outcomes? A study with Spanish and Latin American offenders]. *Psychosoc. Interv.* 24 41–47. 10.1016/j.psi.2015.03.001

[B103] Vives-Cases C. Torrubiano-Domínguez J. Escribá-Agüir V. Ruiz-Pérez I. Montero-Piñar M. I. Gil-González D. (2011). Social determinants and health effects of low and high severity intimate partner violence. *Ann. Epidemiol.* 21 907–913. 10.1016/j.annepidem.2011.02.003 21440455

[B104] Voith L. A. (2017). Understanding the relation between neighborhoods and intimate partner violence: an integrative review. *Trauma Violence Abuse* 10.1177/1524838017717744 [Epub ahead of print]. 29333974

[B105] Waltermaurer E. (2012). Public justification of intimate partner violence: a review of the literature. *Trauma Violence Abuse* 13 167–175. 10.1177/1524838012447699 22643069

[B106] Wee S. Todd M. J. Oshiro M. Greene E. Frye V. (2016). Modifiers of neighbors’ bystander intervention in intimate partner violence: a concept mapping study. *Violence Gender* 3 55–63. 10.1089/vio.2015.0012 27626038 PMC4997712

[B107] World Health Organization [WHO] (2002). *World Report on Violence and Health.* Geneva: World Health Organization.

[B108] World Health Organization [WHO] (2013). *Global and Regional Estimates of Violence Against Women: Prevalence and Health Effects of Intimate Partner Violence and Non-Partner Sexual Violence.* Geneva: World Health Organization.

[B109] Wright E. M. Benson M. L. (2011). Clarifying the effects of neighborhood context on violence “behind closed doors”. *Just. Q.* 28 775–798. 10.1080/07418825.2010.533687

