# Supplementary material for: The Willingness to Intervene in Cases of Intimate Partner Violence Against Women (WI-IPVAW) Scale: Development and Validation of the Long and Short Versions
Source: Front Psychol. 2018 Jul 17;9:1146. doi: 10.3389/fpsyg.2018.01146 (PMC6056762; doi:10.3389/fpsyg.2018.01146)
Supplement: Supplementary file 1 [file Data_Sheet_1.docx]

**Appendix 1**. Items of the long form version form of the WI-IPVAW scale. *nine-item WI-IPVAW short form version. + five-item WI-IPVAW short form version

1. If I was in a bar and a couple that clearly had been drinking too much started insulting and pushing each other, I would ignore them and mind my own business.

*2. If I heard a man shouting violently at his partner in the communal area of my building, I would intervene to stop the situation.

3. If a man insulted his partner on the street, I would say something to express my disapproval.

4. If a woman in a shop said she was afraid because her partner had threatened her, I would advise her to call the police.

5. If a woman knocked on my door to ask for help because her husband has threatened to hit her, I would call the police.

6. If a woman was running away from her partner in the street, I would stop the man.

7. In a supermarket, if a man insulted his wife, I would ignore the situation.

+*8. If I found out that a woman neighbor of mine had been beaten by her husband, I would advise her to report it.

+*9. In a bar, if a man started screaming at his partner, I would stand between them to help the woman.

+*10. If I found out that a woman in my neighborhood was frequently beaten by her partner, but did not want to report it to the authorities, I would call the police.

11. In the street, if a man took his partner’s cell phone and threw it on the ground, I would approach the man and reprehend him for his action.

+*12. In the place where I live, if I overheard a man shouting and threatening his partner, I would go and try to protect the woman.

13. If a woman came into a local bakery, crying and saying that her partner was following her and threatening her, I would call the police.

14. If a couple in the neighborhood was arguing and screaming during the night, I would go and knock on the door to see what was happening.

*15. If a couple started arguing and the man pushed the woman at a bus stop, I would look the other way and ignore the situation.

*16. If a young couple was shouting and insulting each other on the street, I would ignore them.

17. On the staircase or in a communal area where I live, if a woman was asking for help because her partner was hitting her, I would call the police.

18. If a couple was insulting and threatening each other on the street and started to hit each other, I would call the police.

19. If a fierce argument broke out between a couple in a bar in the neighborhood, and both were shouting and insulting each other, I would reprehend them.

20. If a man in the street pushed his partner to the ground, I would intervene and try to stop him.

21. If a woman neighbor said that her husband was threatening her because she wanted to leave him or get a divorce, I would advise her to go to the police.

22. If a woman had been beaten in a bar by her partner and did not want to report it to the authorities, I would call the police.

23. If a couple was having a fierce argument in a local store, I would say something to them to stop the situation.

24. If I overheard fierce arguments and shouting between a couple in the neighborhood, I would ignore it.

25. In a bar, if a man hit his partner during an argument and immediately afterwards asked her to forgive him, I would ignore it.

*26. In a bar, if a woman said her partner had hit her, I would advise her to call the police.

+*27. If an immigrant couple or a couple from another culture were fighting on the street, I would ignore it and keep walking.

28. In a bar. if a couple who had clearly been drinking too much started to push and hit each other, I would call the police.

**Appendix 2.** Scoring the WI-IPVAW

The WI-IPVAW scale has one general factor (i.e., “willingness to intervene”), comprising all items, and three specific factors (i.e., “calling the cops”, “not my business”, and “personal involvement”), comprising different subsets of items. Both the general and specific factors could be used to obtain complementary information. However, if the researcher is interested in the common elements of the measured construct, then the general factor should be used. On the other hand, if the researcher is more interested in the type of interventions proposed by the items, then they should look at the specific factors.

To compute the scores of the scale we do not recommend using the sum of the direct scores of the items, as this procedure assumes that all items are equally important to measure the factors of the scale (i.e., they are tau equivalent); moreover, it could increase the residual correlations between the factors. In addition, caution should be taken with items 6 and 20 of the scale, which have different threshold parameters for men and women. To score the WI-IPVAW scale, we recommend using one of the following procedures:

Estimate the factor scores using the invariant items as anchor items: all the items of the scale are used to compute the factor scores for men and women. In order to make the factor scores comparable across genders, the invariant items are used as anchor items, constraining the loadings and thresholds parameters to be equal across gender groups, whereas the thresholds of the non-invariant items are allowed to vary. We provide an Mplus syntax to estimate this model and obtain the factor scores for men and women in the same metric.

Estimate the factor scores using only the invariant items. Alternatively, the non-invariant items can be removed and the same factorial model estimated for men and women.

**Mplus syntax**

TITLE: Multigroup CFA WI-IPVAW

DATA:

! choose data extension and format. It should include the items and one grouping variable

FILE IS wi-ipvaw.dat; ! insert data name

VARIABLE:

NAMES ARE gender wi1-wi28;

USEV ARE wi1-wi28;

CATEGORICAL = wi1-wi28;

GROUPING IS gender (0=Women 1=Men); ! insert grouping values according to data

ANALYSIS:

ESTIMATOR = WLSMV;

PARAMETERIZATION=THETA;

MODEL:

! factor loadings constrained to be equal across groups

Call BY wi4* wi5 wi8 wi10 wi13 wi17 wi18 wi21 wi22 wi26 wi28 (lcall1-lcall11);

Ignore BY wi1* wi7 wi15 wi16 wi24 wi25 wi27 (lign1-lign7);

PInvol BY wi2* wi3 wi6 wi9 wi11 wi12 wi14 wi19 wi20 wi23 (lpinvol1-lpinvol10);

WI By wi1-wi28*(lwi1-lwi28);

! factor variances fixed to 1 in the reference group

Call@1;

Ignore@1;

PInvol@1;

WI@1;

! correlations between factors fixed to 0

Call WITH Ignore@0;

Call WITH PInvol@0;

Ignore WITH PInvol@0;

WI WITH Call@0;

WI WITH Ignore@0;

WI WITH PInvol@0;

! residuals fixed to 1

wi1-wi28@1;

! latent means fixed to 0 in the reference group

[Call@0]

[Ignore@0]

[PInvol@0]

[WI@0]

MODEL Men:

! factor loadings constrained to be equal across groups

Call BY wi4* wi5 wi8 wi10 wi13 wi17 wi18 wi21 wi22 wi26 wi28 (lcall1-lcall11);

Ignore BY wi1* wi7 wi15 wi16 wi24 wi25 wi27 (lign1-lign7);

PInvol BY wi2* wi3 wi6 wi9 wi11 wi12 wi14 wi19 wi20 wi23 (lpinvol1-lpinvol10);

WI By wi1-wi28*(lwi1-lwi28);

! factor variances free

Call*;

Ignore*;

PInvol*;

WI*;

! correlations between factors fixed to 0

Call WITH Ignore@0;

Call WITH PInvol@0;

Ignore WITH PInvol@0;

WI WITH Call@0;

WI WITH Ignore@0;

WI WITH PInvol @0;

! free thresholds for items 6 and 21

[wi6$1-wi6$5*];

[wi20$1-wi20$5*];

! residuals fixed to 1

wi1-wi28@1;

! free latent means

[Call*]

[Ignore*]

[PInvol*]

[WI*]

OUTPUT:

STDYX;

SAVEDATA: FILE IS WI_fscores.dat; ! return a .dat file with the factor scores

SAVE IS fscores;

FORMAT IS free;
